# Pediatric Penetrating Neck Injury Complicated by Significant Subcutaneous Emphysema in Rural Australia

**DOI:** 10.7759/cureus.119445

**Published:** 2026-11-10

**Authors:** Meg Wood, Abbie Maggs, Nicole Carmody

**Affiliations:** 1 Emergency Department, Mount Isa Base Hospital, Mount Isa, AUS; 2 Anesthesiology Department, Mount Isa Base hospital, Mount Isa, AUS

**Keywords:** airway access, child trauma, pediatric ent, penetrating neck injuries, rural emergency medicine

## Abstract

Although uncommon in the pediatric population, penetrating neck injuries (PNIs) in children carry a high risk of morbidity and mortality. This is due to the concentration of major blood vessels and organs within the neck, making them vulnerable to severe injury even when external wounds appear minor. Management strategies have evolved from traditional mandatory surgical exploration to a more selective approach based on clinical assessment and imaging. Here, we present the case of a three-year-old boy from a remote and rural region of Australia who sustained a small penetrating injury to zone I of the anterior neck. Despite being clinically stable at initial presentation, he rapidly deteriorated, developing significant and expanding subcutaneous emphysema. The patient was successfully managed with airway stabilization via fiberoptic bronchoscopy and transfer to a tertiary center for surgical exploration. This case emphasizes the unique challenges of managing PNIs in rural and remote health care settings, where care is complicated by geographical isolation, limited access to specialist services, and the need to achieve clinical stability before aeromedical retrieval. It also highlights the importance of continuous monitoring and reassessment to recognize clinical deterioration in these high-risk patients.

## Introduction

Penetrating neck injuries (PNIs) are defined as wounds that breach the platysma muscle within the anterior compartment of the neck [1]. These injuries account for 5-10% of all trauma cases and often result from sharp objects, such as knives, glass or metal fragments, and firearms [2]. Although uncommon in the pediatric population, these injuries represent potentially life-threatening emergencies due to the high concentration of vital neurovascular and aerodigestive structures within a confined anatomical region. PNIs in children under the age of 15 years carry a reported mortality rate of 5.6% [3]. Pediatric patients are particularly vulnerable to severe injury because traumatic forces are distributed over a smaller body surface area, resulting in greater force per unit area [4]. Consequently, penetrating trauma, even when associated with minor external wounds, can lead to disastrous complications, including major vascular injury, cervical spine fractures, and severe airway compromise [5]. Symptoms such as an altered Glasgow Coma Scale score, major hemorrhage (including significant hemoptysis or hematemesis), large pulsatile hematomas, and significant subcutaneous emphysema are associated with underlying vascular or aerodigestive injuries [6]. Notably, extensive or expanding subcutaneous emphysema is associated with an 80% incidence of underlying injury to the trachea, hypopharynx, or esophagus [7]. Therefore, clinicians must remain vigilant when evaluating and managing pediatric PNIs.

With almost one-third of Australians living outside major cities [8], potential barriers to accessing healthcare must be considered, particularly for acutely unwell or high-risk patients, such as those with PNIs. Rural communities frequently experience geographical isolation, with many located far from urban healthcare hubs and tertiary facilities. Consequently, these communities may lack the resources to manage specialist emergencies, necessitating the aeromedical retrieval of patients to the nearest tertiary hospital. A study conducted in Western Australia (n = 3,333) found that patients with major trauma in rural areas had more than twice the risk of death compared with those in urban areas. However, when patients were efficiently stabilized and retrieved to an urban facility by the Royal Flying Doctor Service (RFDS), their mortality outcomes were equivalent to those of patients in metropolitan areas [9]. This finding reinforces the need for early recognition and intervention in deteriorating trauma patients, alongside timely transfer to tertiary facilities for life-saving specialist care. A three-year prospective observational study conducted by the Queensland Section of the RFDS in Australia reported a successful in-flight intubation rate of 94.7% [10]. Despite these favorable findings, aeromedical retrieval has limitations, including restricted staffing, limited access to patients during transport, and unpredictable flight conditions. Therefore, establishing a secure and reliable airway before transfer is preferable, as this may minimize the need for emergency interventions during transport.

This case report describes a PNI in a three-year-old boy from a remote rural region of Australia. Although the literature frequently focuses on airway management, particularly intubation techniques and surgical intervention, the educational value of this case extends beyond these aspects. It highlights the importance of maintaining a high index of suspicion for occult injuries despite initial clinical stability, anticipating rapid airway deterioration, and navigating the complexities of trauma management while considering the challenges of aeromedical retrieval in a resource-limited setting.

## Case presentation

A three-year-old boy presented to the emergency department (ED) at Mount Isa Base Hospital (MIBH) approximately 40 minutes after falling from a trampoline. He fell forward onto a metal star picket, sustaining a puncture wound to the anterior neck (Figure 1). His mother reported initial bubbling from the wound, accompanied by a whistling sound during inspiration. There was no reported loss of consciousness, and he cried immediately after the incident. He was reportedly well before the injury, with no significant past medical history, and his immunizations were up to date.

**Figure 1:**
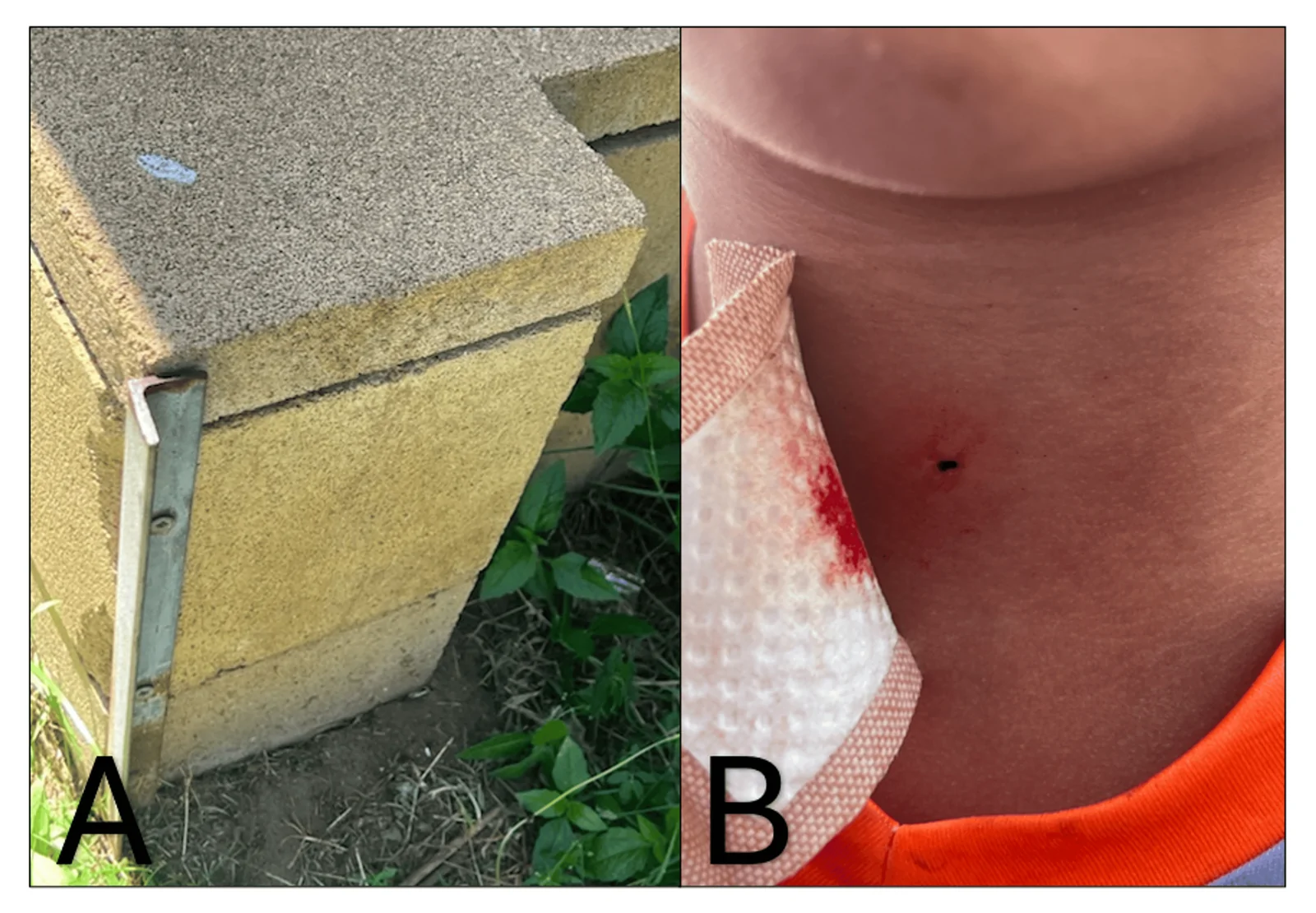
(A) Metal star picket. (B) Puncture wound to the anterior neck

On arrival at the ED, the child was settled and showed no clinical signs of respiratory distress. All vital signs were within normal age-adjusted limits: heart rate, 99 bpm; blood pressure, 104/78 mmHg; respiratory rate, 24 breaths per minute; and oxygen saturation, 99% on room air. He was afebrile. Examination revealed an approximately 0.5-mm-wide puncture wound in the anterior neck, just left of the midline. The base of the wound could not be adequately visualized.

Non-contrast CT of the neck demonstrated pneumomediastinum, with extensive gas tracking through the soft tissues surrounding the visceral space and extending superiorly to the skull base. At the level of the T2 vertebral body, a subtle outpouching of the left anterolateral tracheal wall was observed (Video 1, axial slices 142-144 of 163), which was reported as highly suggestive of direct tracheal injury. The remaining tracheal contours were unremarkable. An incidental small left-sided pneumothorax was also identified.

**Video 1:** Plain CT neck of the three-year-old boy Axial CT images of the patient's neck. In axial slices 142-144 of 163, the red arrow indicates a focal outpouching of the tracheal wall at the level of the T2 vertebral body, highly suggestive of tracheal injury. The axial slice number is displayed within the yellow box. CT: computed tomography

The patient was discussed with the Ear, Nose, and Throat (ENT) team at Townsville University Hospital (TUH), who recommended applying a non-occlusive dressing, transferring the patient at low altitude, and maintaining a low threshold for endotracheal intubation should his clinical status deteriorate. As he was clinically stable at the time, transfer was planned without intubation.

While awaiting transfer via the RFDS, the patient initially underwent observations at 15-minute intervals for the first hour, followed by 30-minute intervals for the next 1.5 hours and hourly intervals thereafter. During this period, he remained stable and asymptomatic. He received 550 mg (25 mg/kg) of intravenous Augmentin for infection prophylaxis.

At approximately six hours and 40 minutes after arrival at the ED, the patient deteriorated. He became distressed, dyspneic, and tachycardic, with a heart rate of 120 bpm, and developed rapidly expanding subcutaneous emphysema. The emphysema was extensive, with visible expansion of the face, neck, and chest wall (Figure 2). His eyes became swollen shut, and his oxygen saturation dropped to 94%. His last recorded observations before this deterioration had been stable (respiratory rate, 15 breaths per minute; oxygen saturation, 99% on room air; and heart rate, 84 bpm). The initial impression was that the clinical deterioration was due to pain from the subcutaneous emphysema; however, the cause of its sudden and marked progression was unclear. The patient was treated with 100% oxygen via a non-rebreather (NRB) mask and 20 micrograms (mcg) of IV fentanyl. Following fentanyl administration, he developed localized urticaria on his abdomen, for which 200 mcg of intramuscular adrenaline was administered, with good effect.

**Figure 2:**
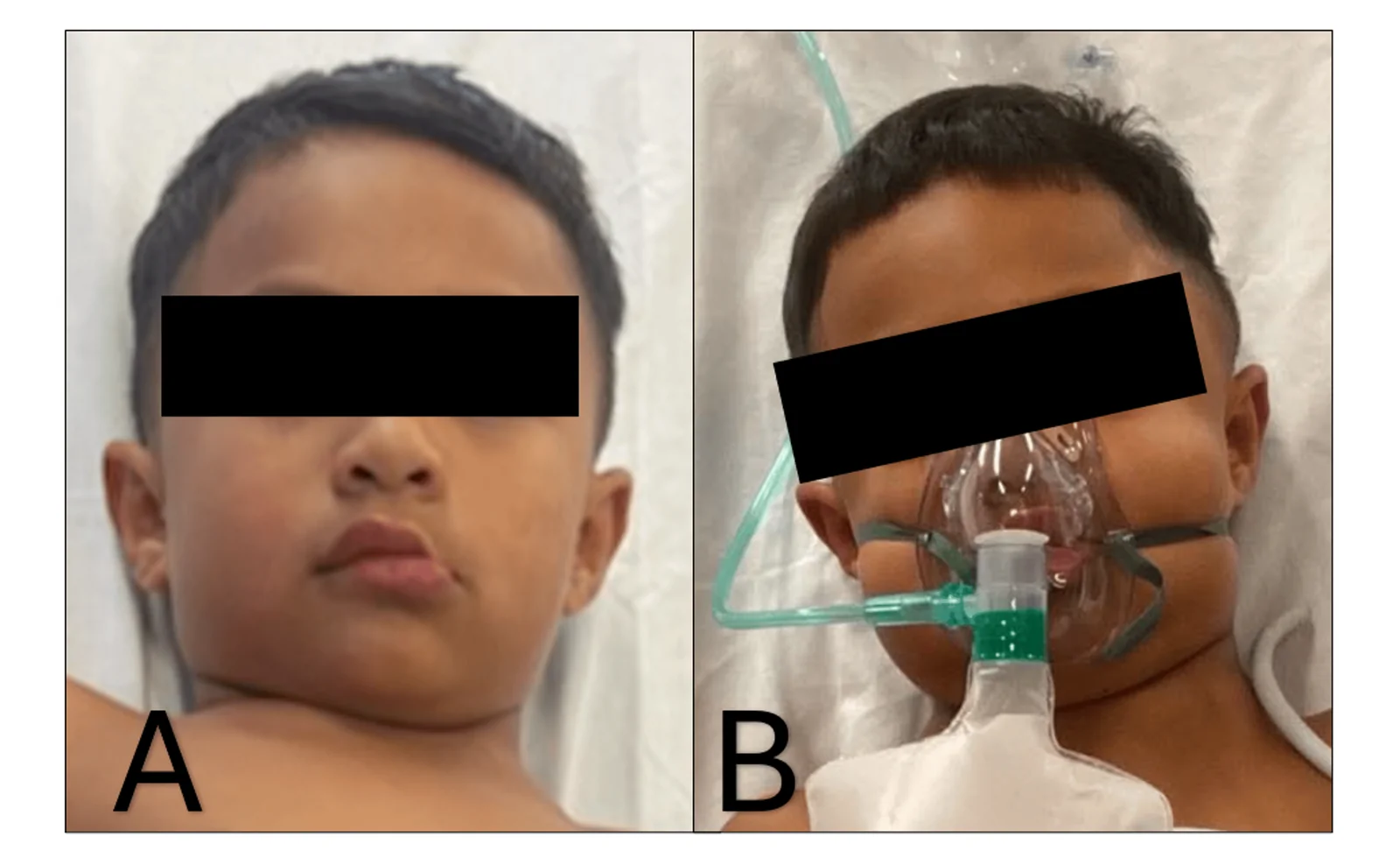
(A) Patient's appearance on arrival at the ED. (B) Patient's appearance following clinical deterioration and the development of extensive subcutaneous emphysema ED: emergency department Written informed consent from the patient's legal guardian, permitting the disclosure of the patient's identifying information in an open-access publication, was provided to the journal.

In view of the rapidly progressive subcutaneous emphysema and concern for impending airway compromise, the on-call anesthetist was contacted but was unable to attend due to ongoing anesthetic responsibilities in the operating theater. The second on-call anesthetist, a rural generalist, was able to attend. In anticipation of severe airway compromise, the clinical team prepared for emergency rapid sequence induction (RSI) and endotracheal intubation, with a view to performing an emergency tracheostomy in the event of cardiac arrest. However, the patient stabilized. His airway remained patent, his oxygen saturation remained above 94% while receiving oxygen at 15 L/min via an NRB mask, and he maintained good bilateral air entry without stridor on serial examinations. Given the risks associated with emergency intubation, the team decided to continue observation without intubation at that time.

Upon arrival of the pediatric intensive care unit (PICU) retrieval team, the patient was taken to the operating theater for a planned inhalational induction to maintain spontaneous breathing and facilitate visualization of the trachea using fiberoptic bronchoscopy. Bedside ultrasonography was performed to assess the size of the left pneumothorax previously identified on CT. A small pneumothorax was confirmed, but no intervention was required. During inhalational induction, the patient experienced partial laryngospasm, resulting in airway obstruction. Consequently, the approach was changed to RSI, which resolved the obstruction. The patient successfully underwent fiberoptic bronchoscopy-assisted endotracheal intubation, with no tracheal defect visualized. He was transferred to TUH on a low-altitude flight 10.5 hours after his initial presentation to the MIBH ED.

At TUH, CT angiography of the neck revealed a PNI with a dominant full-thickness midline laceration extending to the anterior wall of the trachea. The scan also demonstrated interval progression of the subcutaneous emphysema, which had dissected through essentially all visualized cervical and deep neck fascial planes, extending into the superior mediastinum and resulting in moderate-to-large-volume pneumomediastinum. No vascular injury was identified. The patient underwent surgical exploration of the neck wound the following day, which revealed a left paratracheal injury without direct tracheal involvement.

He was successfully extubated on postoperative day 2. Diagnostic bronchoscopy performed immediately after extubation revealed no tracheal injury or deformity. A follow-up barium swallow study was normal. By day 6 post-injury, the subcutaneous emphysema had significantly improved, with only minimal residual emphysema persisting inferior to the right clavicle.

His outpatient recovery was complicated by wound dehiscence and a superficial surgical site infection, which were successfully managed with a five-day course of oral cefalexin (12.5 mg/kg every six hours). At follow-up 26 days after the injury, his mother reported that he was doing very well, had returned to his usual activities, and that the wound had fully healed.

## Discussion

PNIs present a range of diagnostic and management challenges that are amplified in rural and remote regions. This case highlights the challenges of managing major trauma in a remote region of Australia, where the nearest ENT and tertiary trauma center is approximately 560 miles away, equivalent to a 10-hour drive or a 140-minute flight. Such geographical isolation introduces significant logistical challenges and can delay access to definitive care. MIBH, where the child initially presented, is a Level II trauma center in Queensland. One study found that between 2018 and 2024, MIBH received a total of 208 tier 1 trauma cases. Of these, 39.9% resulted from motor vehicle accidents, 23.6% from assaults, and 27.3% from mechanical falls. The most commonly injured anatomical regions were the chest (40.4%) and head and neck (39.4%). Approximately 51% of patients required aeromedical transfer to tertiary centers, while 29.8% underwent surgical interventions by local specialists. Although 90-day mortality was comparable to national benchmarks at 7.2%, trauma care remains dependent on critical stabilization and damage control at rural facilities, followed by timely retrieval to a tertiary center [11].

When evaluating and managing PNIs, the neck is traditionally divided into three anatomical zones. This zonal classification has important implications for clinical assessment, diagnostic evaluation, and surgical management [12]: Zone I extends from the clavicles to the level of the cricoid cartilage and includes structures such as the trachea, esophagus, thyroid gland, subclavian artery, and proximal common carotid artery; Zone II extends from the cricoid cartilage to the angle of the mandible and includes structures such as the trachea, esophagus, pharynx, larynx, vagus nerve, and jugular vein; and Zone III extends from the angle of the mandible to the base of the skull and includes structures such as the distal carotid artery, distal jugular vein, cranial nerves, salivary glands, and parotid glands. The vertebral arteries and spinal column traverse all three zones [13].

Historically, the management of PNIs was guided by the anatomical zone involved and the mechanism of injury. Zone II is the most commonly injured region, and injuries in this zone may have serious consequences in pediatric patients. This is due to their smaller anatomical dimensions, which result in the close proximity of vital structures, including the esophagus, airway, and major blood vessels [12]. Zone II injuries were previously managed with mandatory surgical exploration, whereas injuries involving Zones I and III were assessed based on clinical findings and hemodynamic stability. This approach has since evolved, with the Western Trauma Association developing an algorithm for the management of PNIs [14]. This algorithm aligns with the Advanced Trauma Life Support protocol and incorporates hemodynamic stability alongside the presence of hard and soft signs of injury (Figure 3). It advocates a selective approach to managing penetrating Zone II neck injuries, emphasizing CT-based assessment and careful clinical evaluation [14]. This approach facilitates the identification of patients who require operative intervention while reducing unnecessary surgery and its associated morbidity. However, it is important to acknowledge the limitations of applying predominantly adult-derived trauma management guidelines to the pediatric population.

**Figure 3:**
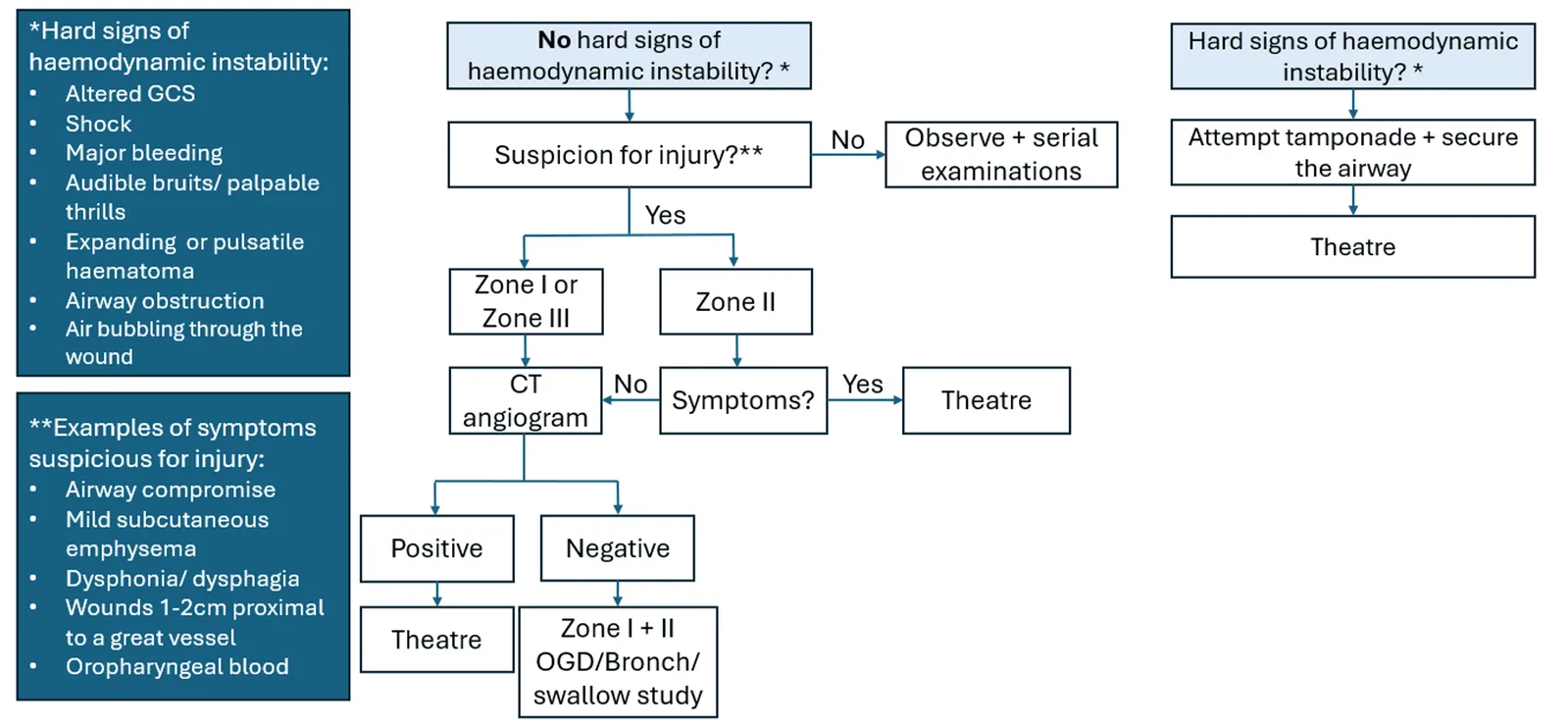
Revised management of PNIs The proposed management algorithm is adapted from the Western Trauma Association guidelines for the management of PNIs [14] and incorporates both hard and soft signs of injury [6]. PNIs: penetrating neck injuries

The patient in this case sustained a Zone I injury at the T2 vertebral level. In accordance with the Western Trauma Association guidelines, the patient's management was guided by imaging findings and the evolving clinical picture. The patient showed no signs of vascular injury but demonstrated both hard and soft signs of aerodigestive tract injury, including air bubbling through the wound and subcutaneous air on CT. The outpouching of the anterior tracheal wall on CT was highly suggestive of direct tracheal injury. Accordingly, the patient was planned for aeromedical transfer to a specialist ENT department for further evaluation and consideration of surgical exploration.

In this case, the child had to wait in Mount Isa for retrieval by the RFDS, during which time his condition deteriorated. A key question arising from this case is whether earlier intervention and airway management could have prevented the subsequent clinical deterioration. This is difficult to determine given the patient's initial presentation. On arrival at the ED, he was clinically stable. It was only when the child became distressed and agitated that the subcutaneous emphysema began to expand and track superiorly into the face. It is possible that early intubation could have reduced the risk of subsequent clinical deterioration; however, this remains uncertain given the patient's period of stability and reassuring initial clinical presentation. Furthermore, earlier intubation could have introduced additional risks associated with airway instrumentation in the setting of suspected tracheal injury. Consequently, it is difficult to determine whether earlier intervention would have altered the patient's clinical course.

The patient was initially planned for transfer without intubation or mechanical ventilation. However, following the rapid progression of subcutaneous emphysema and increasing concern for impending airway obstruction, the risk of deterioration during transport became unacceptably high. Therefore, it was decided that endotracheal intubation before transfer was in the patient's best interest. This decision presented an additional challenge in the remote ED, where clinicians have limited opportunities to perform advanced airway management in pediatric patients. Consequently, airway interventions in critically ill children require careful planning, clear communication, and consideration of the resources available should complications arise. Failed or repeated intubation attempts may force air through injured tissues and fascial planes, causing further airway trauma and potentially worsening airway obstruction [15]. Additionally, positive-pressure ventilation (PPV) proximal to the level of injury during RSI, or resulting from incorrect endotracheal tube placement, may further exacerbate subcutaneous emphysema and lead to clinical deterioration [16,17]. One approach to overcoming this challenge in stable patients with suspected or confirmed tracheal injury is inhalational induction with fiberoptic bronchoscopy-assisted intubation [17,18]. Inhalational induction is preferable in these cases because it maintains muscle tone and spontaneous ventilation, allowing for a controlled setup and guided intubation without the need for PPV. In contrast, emergency intubation is a major risk factor for tracheal rupture. Although it remains the method of choice in emergency situations, the associated risk of death has been reported to be three times higher than that of elective intubation [17,19]. When available, fiberoptic bronchoscopy-assisted endotracheal intubation should be considered because it allows direct visualization of the airway during intubation [20]. Additionally, this technique can be performed with the patient awake when necessary, making it a particularly useful option when full sedation is contraindicated or otherwise inadvisable [20]. In this case, the patient's airway remained stable until he was transferred to the operating theater for planned fiberoptic bronchoscopy-assisted endotracheal intubation.

An important consideration in this case is the workforce limitations frequently encountered at rural hospitals. This incident occurred during daytime hours on a weekend, when staffing arrangements provided access to two on-call anesthetists. This provided an additional level of anesthetic support. Although competing responsibilities in the operating theater prevented the first on-call anesthetist from attending, a second on-call anesthetist, a rural generalist with anesthetic training, was available. Due to staffing limitations at this rural hospital, had this incident occurred on a weekday, only one anesthetist would have been on call, leaving no additional anesthetically trained clinicians available to assist in the ED. Furthermore, there is no on-site anesthetic coverage outside regular hours. This highlights the challenges associated with limited staffing in rural hospitals, where reduced access to additional clinicians can significantly restrict the availability of support and backup during critical emergencies.

A further consideration is that the patient needed to be clinically stable enough to tolerate aeromedical transfer. RFDS retrieval teams typically consist of one doctor and one nurse, meaning that patients must be sufficiently stable to be safely managed during flight with limited personnel and resources. The PICU retrieval team that arrived consisted of one doctor and one nurse redeployed from the TUH PICU department. As the team primarily comprised ward-based clinicians rather than retrieval specialists, their involvement highlights a key challenge in aeromedical retrieval: the availability of dedicated retrieval personnel to undertake interhospital transfers. Despite this limitation, the team successfully maintained the patient's clinical stability throughout the transfer. Additionally, due to the patient's subcutaneous emphysema, he required transfer at a low altitude. At high altitudes, atmospheric pressure decreases, potentially causing trapped air to expand and worsening subcutaneous emphysema [21]. Even minor subcutaneous emphysema can become dangerous at high altitudes if the underlying cause remains unresolved [21].

Lastly, it is important to discuss the discrepancy between the suspected tracheal injury on imaging and the absence of a confirmed tracheal defect during bronchoscopy. The child's initial clinical presentation, together with the original CT findings, was highly suggestive of tracheal injury. Management was guided by the clinical picture and undertaken in accordance with pediatric PNI guidelines. Interestingly, surgical exploration revealed a left paratracheal injury, while direct visualization via bronchoscopy showed no evidence of direct tracheal injury. It is possible that the patient did not have a direct tracheal injury. The expanding subcutaneous emphysema may have resulted from increased intrathoracic pressure generated during active crying, potentially forcing air through the fascial planes. This may also explain why the patient's condition stabilized when he became less distressed following analgesic administration. Alternatively, both traumatic [22] and nontraumatic tracheal injuries [23] have been reported to resolve with conservative management. The possibility of a small tracheal injury that spontaneously resolved before bronchoscopy on day 3 post-injury cannot be entirely excluded, although this is considered less likely.

## Conclusions

PNIs in pediatric patients can result in significant morbidity and potentially life-threatening airway compromise, even when the initial presentation is reassuring. This case highlights the importance of maintaining a high index of clinical suspicion for aerodigestive injury, as well as the need for repeated clinical assessments and close monitoring to promptly identify deterioration. When tracheal injury is suspected, airway management requires careful consideration of the risks associated with both delayed intervention and instrumentation of a potentially injured airway. Preparedness for the rapid deterioration of initially stable patients is essential, and clinicians must be familiar with the available intubation techniques.

These challenges are compounded in remote healthcare settings, such as rural Australia, where geographical isolation, limited access to specialist services, and variable anesthetic staffing may constrain available management options. Furthermore, the need to achieve clinical stability before aeromedical retrieval introduces an additional consideration when planning definitive care.
